# Giant Stress Impedance Magnetoelastic Sensors Employing Soft Magnetic Amorphous Ribbons

**DOI:** 10.3390/ma13092175

**Published:** 2020-05-08

**Authors:** Juan Jesús Beato-López, Juan Garikoitz Urdániz-Villanueva, José Ignacio Pérez-Landazábal, Cristina Gómez-Polo

**Affiliations:** 1 Departamento de Ciencias, Universidad Pública de Navarra, 31006 Pamplona, Spain; urdaniz.106234@e.unavarra.es (J.G.U.-V.); ipzlanda@unavarra.es (J.I.P.-L.); 2 Institute for Advanced Materials and Mathematics INAMAT^2^, Universidad Pública de Navarra, 31006 Pamplona, Spain

**Keywords:** soft magnetic amorphous alloys, magnetoelastic coupling, magnetostriction, magnetoelastic sensors, GSI effect, cross-section characterization, liquid flow meter sensor

## Abstract

Soft magnetic amorphous alloys obtained via rapid quenching techniques are widely employed in different technological fields such as magnetic field detection, bio labeling, non-contact positioning, etc. Among them, magnetoelastic applications stand out due to excellent mechanical properties exhibited by these alloys, resulting from their amorphous structure, namely, their high Young modulus and high tensile strength. In particular, the giant stress impedance (GSI) effect represents a powerful tool to develop highly sensitive magnetoelastic sensors. This effect is based on the changes in the high-frequency electric impedance as the result of the variation in magnetic permeability of the sample under the action of mechanical stresses. In this work, the GSI effect is analyzed in two soft magnetic ribbons ((Co_0.93_ Fe_0.07_)_75_ Si_12.5_ B_12.5_ and (Co_0.95_ Fe_0.05_)_75_ Si_12.5_ B_12.5_) for the subsequent development of two practical devices: (i) the characterization of the variations in the cross-section dimensions of irregularly shaped elements, and (ii) the design of a flow meter for measuring the rate of flow of water through a pipe.

## 1. Introduction

Rapid quenching techniques enable the fabrication of soft magnetic amorphous alloys with a wide range of compositions and different shapes. These alloys are extensively applied in different technological fields and commercial applications such as transformer cores, magnetic field sensors, motors, electric vehicles, non-contact positioning, bio labeling, biosensors (food safety, medical applications), etc. [1,2]. Their extensive use is due to their unique properties resulting from their amorphous structure. The lack of magnetocrystalline anisotropy and structural defects confers them excellent soft magnetic properties that can be easily tailored during the fabrication procedure and with subsequent magnetic, mechanical, or thermal treatments [3]. In addition to that, these alloys show optimal chemical, mechanical, and magnetoelastic properties. including resistance to corrosion, high tensile stress and Young modulus, uniformity, thin shapes, low size (enabling miniaturization and high-frequency devices), and high magnetic permeability [4,5]. The combination of all these properties turns these alloys into suitable materials for the design of different kinds of sensors with high sensitivity, low cost, and quick response [6].

Focusing on magnetoelastic sensors, different applications, geometries, and compositions were reported [7,8]. The magnetoelastic anisotropy facilitates the control of the magnetization state through the magnetoelastic coupling between the magnetization and the mechanical stresses applied externally, *σ*. This phenomenon is basically governed by the magnetostriction, *λ_s_*, of the sample. Basically, two different ranges can be defined in rapidly quenched amorphous alloys, considering those mainly composed of 70–80% metal transition elements (Fe, Co) and 30–20% of metalloid elements (Si, B): highly magnetostrictive ($\lambda_{s}≳\;10^{-6})$ and nearly zero magnetostrictive alloys (*λ_s_* ~ 10^−7^). In the first case, both positive (Fe-based) and negative (Co-based) magnetostrictive alloys can be found, showing a very sensitive response to the applied stress and experiencing large elongations in their size [8,9]. However, for Co-rich alloys with a small percentage of Fe (~5%) nearly zero magnetostriction values are obtained with even softer magnetic properties [8]. 

However, despite the low magnetostriction constant, magnetoelastic sensors with vanishing magnetostriction were reported based on the stress dependence of the giant magnetoimpedance effect (GMI) [10,11]. The GMI effect, mainly employed in the design of highly sensitive magnetic sensors, is characterized by large variations of the high-frequency electric impedance, *Z*, of a soft magnetic material submitted to a direct continuous (*DC*) axial magnetic field, *H*. The changes in *Z* can be explained in terms of the variation of the skin penetration depth, δ=ρπfμ (where ρ is the resistivity, *f* is the frequency of the exciting alternating current (*AC*) in the sample, and *µ* is the transversal magnetic permeability) as a result of the modification of the magnetic permeability when *H* is applied (*μ* = *μ*(*H*)). Accordingly, maximum GMI ratios are achieved in soft magnetic samples easily magnetized under the transverse magnetic field associated with the flow of the electric current (i.e., nearly zero magnetostrictive alloys with negligible *intrinsic* magnetoelastic contribution). Similarly, the giant stress impedance effect (GSI) can be defined [12]. This effect represents a powerful tool for the characterization of the applied mechanical stresses, *σ*, due to the measurable changes in *Z* as a result of stress dependence of the magnetic permeability (*μ* = *μ*(*σ*)). In this case, detectable *Z* variations occur as a result of the changes in *δ* with *σ*. Thus, optimum GSI ratios are obtained as a counterbalance between the negligible magnetoelastic *intrinsic* magnetoelastic anisotropy contribution (low *λ_s_*) to promote the occurrence of skin effect and effective magnetoelastic coupling to give rise to changes in *δ* with *σ*. Consequently, different sensor elements were employed for the design of stresses devices as microwires [13,14], wires [15,16,17], ribbons [18,19,20,21], and multilayer structures [22,23,24].

In this work, the GSI response of two nearly zero magnetostrictive amorphous ribbons with nominal composition (Co_0.93_ Fe_0.07_)_75_ Si_12.5_ B_12.5_ and (Co_0.95_ Fe_0.05_)_75_ Si_12.5_ B_12.5_ is analyzed in terms of the effect of the initial magnetoelastic anisotropy and the magnetoelastic coupling when external stresses, σ, are applied. The observed behavior is explained in terms of the features (value and sign) of the vanishing magnetostriction constant, determined through the evolution of the stress dependence of the anisotropy field estimated through the stress GMI response. Finally, the observed properties were applied for the design of two magnetoelastic devices, one for the characterization of the variations in the cross-section of pieces and another for the estimation of the water flow within a pipe.

## 2. Materials and Methods 

Two amorphous ribbons, with nominal composition *P_1_* (Co_0.93_ Fe_0.07_)_75_ Si_12.5_ B_12.5_ (66 µm thickness, 0.40 mm width) and *P_2_* (Co_0.95_ Fe_0.05_)_75_ Si_12.5_ B_12.5_ (80 µm thickness, 0.35 mm width) were obtained through the melt-spinning technique. Firstly, the conditions for optimum GMI effect (maximum impedance variations) were studied. Both ribbons were excited within a voltage divider configuration with an *AC* sinusoidal signal of frequency, *f*, and peak-to-peak current amplitude, *I_pp_*, generated by a standard function generator (DS 345, Standford Research Systems, Sunnyvale, CA, USA). The output voltage changes, $V=ZI_{pp}$ (*Z*: electric impedance), were measured (oscilloscope MDO 3024, Tektronix, Beaverton, OR, USA) as a function of *f* under the effect of a null and a maximum *DC* axial magnetic field, *H*, (*H_MAX_* = 4.5 kA/m), generated by a homemade solenoid. The acquisition process was controlled with LabVIEW 2014 (National Instruments, Austin, TX, USA). Optimal conditions, namely, maximum GMI ratios defined as $\frac{\Delta Z}{Z}\left(\%\right)=\frac{\left|Z\left(H=0\right)\right|-\left|Z\left(H_{MAX}\right)\right|}{\left|Z\left(H_{MAX}\right)\right|}\times 100$, were found at *f* = 600 kHz and *I_pp_* = 69 mA and *f* = 500 kHz and *I_pp_* = 101 mA for *P_1_* and *P_2_* samples, respectively. These conditions were employed in the impedance characterizations unless otherwise stated.

### 2.1. Magnetoelastic Characterization

**Figure 1:**
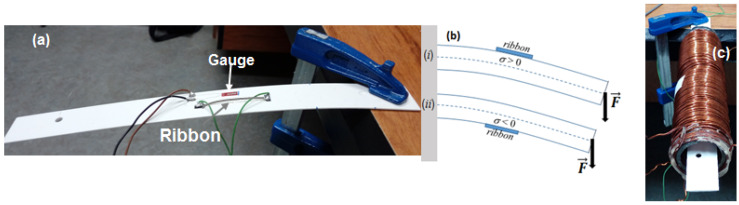
(**a**) Experimental set-up for applying stresses; (**b**) scheme of the applied mechanical stresses to ribbons *P_1_* and *P_2_*; (**c**) experimental set-up for the estimation of the magnetostriction constant.

To characterize the magnetoelastic response of the sample, the evolution of *Z* under tensile and compressive stresses was performed. For this purpose, the magnetoelastic ribbon (5 cm in length) was fixed with an epoxy (Araldite, Ceys, Barcelona, Spain) on the surface of a flat plastic holder (0.2 cm thickness and 30 cm length) close to its middle point (see Figure 1a). One side of the plastic holder was fixed and the other hung up freely, in such a way that, when different loads were added to the free side, controlled mechanical stresses were applied to the sample. Due to the much larger thickness of the plastic support, the neutral axis of the system was placed along the longitudinal axis of the plastic holder (dashed line in Figure 1b) and, thus, the sign of the effective stresses applied to the ribbon was easily controlled. As Figure 1b shows, tensile stresses (*σ* > 0) were applied for a concave curvature and upward position of the ribbon, while compressive stresses (*σ* < 0) were produced for a convex curvature and downward position ((*i*) and (*ii*) in Figure 1b, respectively). The estimation of *σ* was performed through Hooke’s law, *σ* = *E_r_ε_g_*, where *E_r_* = 100 GPa is the Young modulus of the ribbons and *ε_g_* is the effective strain measured by a strain gauge (nominal resistance of 120 Ω and gauge factor 2.1) located close to the middle point of the ribbon (see Figure 1a).

With this experimental system, the variations of impedance (at *H* = 0) as a function of the applied stresses (*Z = Z(σ)*) were acquired. The measured data were normalized by following the expression that defines the giant stress impedance (GSI) ratio, $\frac{\Delta Z_{\sigma }}{Z}\left(\%\right)=\frac{\left|Z\left(\sigma \right)\right|-\left|Z\left(R_{DC}\right)\right|}{\left|Z\left(R_{DC}\right)\right|}\times 100$, where the normalized factor $Z(R_{DC})$ was chosen equal to the *DC* resistance of the samples. 

Furthermore, the designed experimental set-up also enabled the characterization of the magnetostriction constant, *λ_s_*, of the samples. To that effect, the system shown in Figure 1a was introduced in the solenoid (see Figure 1c). It must be pointed out that only small deflections with respect to the solenoid axis should be introduced to ensure a constant *H* along the entire ribbons’ length. As previously reported [25,26], maximum values in *Z* take place for *H* values around the characteristic anisotropy magnetic field, $H_{K}$ ($H_{K}=\frac{2K}{\mu_{0}M_{s}}=\frac{3\lambda_{s}\sigma }{\mu_{0}M_{s}}$, where *μ*_0_ is the vacuum permeability, *M_S_* is the saturation magnetization and $K=\frac{3}{2}\lambda_{S}\sigma$). Thus, the estimation of *H_K_* as a function of *σ* through *Z*(*H*) under applied stress enables the estimation of *λ_s_* of the samples.

### 2.2. Magnetoelastic Sensors

#### 2.2.1. Variations in the Cross-Section Dimensions of Elements

The GSI effect can be employed in the design of a magnetoelastic sensor for the characterization of the dimensions in the cross-section of elements or objects. In this device, the magnetoelastic ribbon is attached (glued) to the perimeter of the element to be characterized. Changes in the cross-section of the analyzed element induce mechanical strains in the glued magnetoelastic ribbon and, accordingly, changes in its electric impedance. Different controlled mechanical tests were performed to simulate the real operation conditions of the potential stress sensor. Firstly, homogeneous cylindrical methacrylate probes of diameters *D_m_* = 20 and 40 mm and length *L_m_* = 5 cm were used. The short length of the probes prevents lateral buckling under the performed compressive tests. Then, the sensor ribbon was fixed tightly to the external perimeter of the methacrylate cylinder using epoxy Araldite. Different ribbon lengths (*L_r_* = 5 and 11 cm) were chosen for covering the whole cylinder perimeter ($L_{r}=\pi$*D_m_*). The probes were submitted to compressive tests (Shimadzu Ag-X-50 testing machine with a 50 kN load cell), where the load, *σ_o_*, was applied to the upper and lower surface of the cylinder. As a result, a decrease in the length of the probes, Δ*L_m_*, took place (measured with a video extensometer), leading to an increment of the cylinder diameter, Δ*D_m_*, with respect to the initial value. Therefore, Δ*D_m_* can be calculated from the experimental parameter Δ*L_m_*, by employing the following expression [19]:

(1) $$\frac{\Delta D_{m}}{D_{m}}=-\upsilon_{m}\frac{\Delta L_{m}}{L_{m}},$$

where $\upsilon_{m}$= 0.40 is the Poisson coefficient of the methacrylate. Since the ribbons were tightly fixed to the cylinder, the variations in diameter (Δ*D_m_*) caused the application of tensile stresses in the ribbons and, consequently, measurable changes in *Z*. For every mechanical test, the obtained *Z* values were normalized using the expression $\frac{\Delta Z_{D}}{Z}\left(\%\right)=\frac{\left|Z\left(D\right)\right|-\left|Z_{MIN}\right|}{\left|Z_{MIN}\right|}\times 100$, where $Z_{MIN}$ is the lowest value of the impedance for each sample and test and Z(D) is the impedance variation as a function of *D_m_*.

Furthermore, the mutual effect of temperature and fixing agent (epoxy or adhesive) in the transmission of mechanical stresses was checked. Therefore, two *P_1_* ribbons with 5 cm length were fixed around the perimeter of a cylindrical probe of *D_m_* = 20 mm employing two different fixing agents: epoxy adhesive (Araldite) and cyanoacrylate. Initially, the probe was introduced in a climate chamber (Binder KBF 115) to study, simultaneously, the evolution of the impedance with both adhesives when the temperature was changed from 5 to 40 °C. Finally, the transmission of mechanical stresses was examined. For that purpose, the same probe was subjected to standard mechanical tests at different temperatures, room temperature (RT = 20 °C) and *T* = 40, 50, and 60 °C. The evolution of the impedance of *P_1_*, $\frac{\Delta Z_{D}}{Z}\left(\%\right)$, was simultaneously registered as a function of the probe diameter with both resins.

#### 2.2.2. Development of a Flow Meter

**Figure 2:**
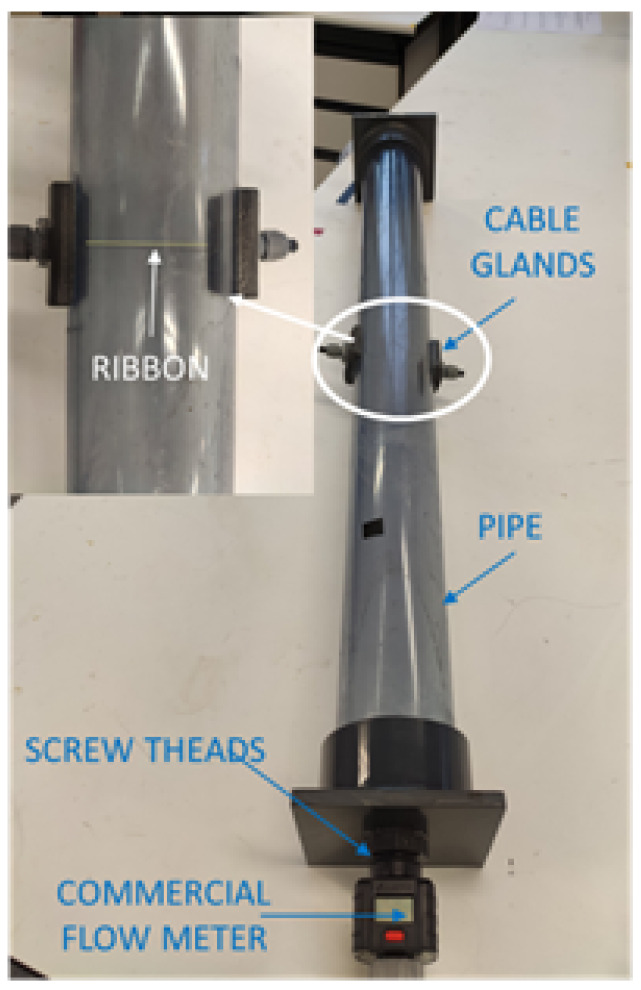
Experimental set-up for the development of water flow meter. The pipe, connections, and position of the ribbon are shown.

The GSI effect of the *P_1_* sample was also employed in the design of a water flow meter. The experimental set-up employed is shown in Figure 2. It consists of a plastic pipe of 9 cm of diameter and 1 m length where the water flows. A magnetoelastic ribbon was perpendicularly fixed to the flow direction using two cable glands. One screw thread per side was installed to connect the pipe to the regular water pipe installation. Because of the conductive nature of water, the employed ribbon (15 cm long) was isolated by using a standard insulator varnish, ensuring that the ribbon was electrically isolated from the water flow. A pre-tightened state in the ribbon was necessary to observe measurable impedance variations. Once the pipe was full of water, the water flow was increased; thus, mechanical (bending) stresses were induced in the sample, leading to measurable changes in *Z*. Simultaneously the flow was measured with a commercial flow meter (Gardena 8188-20, Gardena, Ulm, Germany) installed at the ending side of the pipe. The resulting variations of impedance as a function of the flow, *Z*(*F*), were normalized with the expression $\frac{\Delta Z_{F}}{Z}\left(\%\right)=\frac{\left|Z\left(F\right)\right|-\left|Z(F_{MAX})\right|}{\left|Z(F_{MAX)}\right|}\times 100$, where *Z*(*F_MAX_*) is the value of the impedance when the water flow is maximum.

## 3. Results and Discussion

### 3.1. GSI Response and Magnetoelastic Coupling Analysis

**Figure 3:**
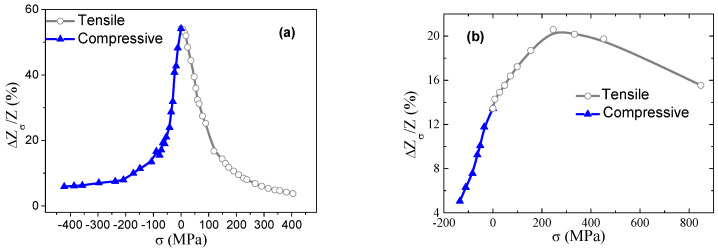
Variation of the giant stress impedance (GSI) ratio $\frac{\Delta Z_{\sigma }}{Z}\left(\%\right)$ with applied mechanical stresses at H = 0 for: (**a**) *P_1_* and (**b**) *P_2_*.

Firstly, the GSI response of the ribbons under axial (tensile and compressive, see Figure 1b) stresses at *H* = 0 was characterized. As can be seen in Figure 3, both samples display a clear different magnetoelastic response. Firstly, the sample *P_1_* shows a maximum for the unstressed state (*σ* = 0 MPa), leading to a clear diminution in *Z* when both tensile and compressive stresses are applied. However, different behavior is observed for *P_2_*, where the maximum impedance does not occur at the unstressed state. Starting from *σ* = 0 MPa, the application of compressive stresses causes a decrease in *P_2_* impedance. On the contrary, the application of tensile stresses leads to an increase of *Z* and, consequently, of the GSI ratio, obtaining its maximum value close to *σ* = 300 MPa. Further application of tensile stresses leads to a large region where GSI ratio slowly decreases until the most intense tensile stress (*σ* = 850 MPa) is applied. This effect can be ascribed to the appearance of internal stresses in the sample during sample fabrication procedure (melting spinning technique) and the occurrence of a different intrinsic magnetoelastic contribution in both samples. 

**Figure 4:**
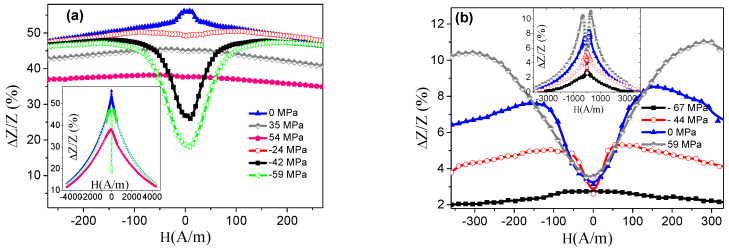
Evolution of the relative variations of impedance, $\frac{\Delta Z}{Z}\left(\%\right)$, with H for samples (**a**) *P_1_*and (**b**) *P_2_*. Samples were excited with a current amplitude of *I_pp_* = 40 mA.

To evaluate this effect, namely, the contribution of the intrinsic magnetoelastic anisotropy, the magnetostriction constant, *λ_s_*, was evaluated in both samples through the analysis of *Z*(*H*) at different applied *σ*. Figure 4 shows the impedance ratio, $\frac{\Delta Z}{Z}\left(\%\right)$, versus *H* as a function of *σ* (compressive and tensile) for the (a) *P_1_* and (b) *P_2_* sample. In this case and similarly to Figure 3, the value of $\frac{\Delta Z}{Z}\left(\%\right)$ was normalized with respect to the *DC* electric resistance, resulting in the expression $\frac{\Delta Z}{Z}\left(\%\right)=\frac{\left|Z\left(H\right)\right|-\left|Z\left(R_{DC}\right)\right|}{\left|Z\left(R_{DC}\right)\right|}\times 100$.

Initially, the sample *P_1_* in the unstressed state undergoes a continuous decrease in *Z* with *H* (see Figure 4a). This GMI behavior is known as single peak (*SP*). The application of compressive stresses (*σ* < 0) promotes the appearance of a double maximum in the impedance curve, known as double peak (*DP*). This impedance evolution indicates a positive value of the magnetostriction constant (*λ_s_* > 0), that is, a reinforcement of the transverse anisotropy field under *σ* < 0. Thus, under the effect of tensile stresses (*σ* > 0), the *SP* behavior remains but a decrease in Z(H) values is detected as a consequence of the reinforcement of the longitudinal easy axis, leading to a decrease in the effective transverse magnetic permeability involved in the GMI effect. Particularly for *H* = 0, a similar trend to that in Figure 3 is obtained, that is, a decrease in *Z* with respect to the unstressed state when tensile or compressive stresses were applied.

On the other hand, the sample *P_2_* exhibits an opposite behavior (see Figure 4b), with the initial unstressed state characterized by a *DP* behavior. In this case, the application of tensile stresses (*σ* > 0) causes the displacement of the maximum of *Z* to higher *H* field values together with an increase in the maximum impedance variations, indicating a negative value of the magnetostriction constant (reinforcement of the transverse anisotropy field under *σ* > 0). An opposite effect is found for compressive stresses (*σ* < 0), namely, the displacement of the maximum toward lower *H* field values and a progressive decrease in the maximum impedance variations. Again, at *H* = 0, the results are consistent with Figure 3, where an increase in *Z* is produced with respect to the unstressed state when axial tensile stresses were applied (and vice versa, decrease in Z for *σ* < 0).

The actual value of $\lambda_{s}$ can be calculated from the evolution of the estimated $H_{K}$ through the axial *H* at which Z displays a maximum value. It is important to note that the components of the impedance, *Z = R + jX*, (with *R* and *X* the real and imaginary part) evolve differently with *I_pp_*. While the maximum in the curve *X(H)* stays nearly constant around $H_{K}$, independently of the value of *I_pp_*, a dependence is found for *R(H)* curve [25]. Since, in this study, only the modulus of *Z* was measured, the samples (see Figure 4) were excited at lower electric current amplitude (*I_pp_* = 40 mA) to assure a low-exciting transverse magnetic field and, therefore, according to the literature, an accurate estimation of $H_{K}$ through this procedure [25].

**Figure 5:**
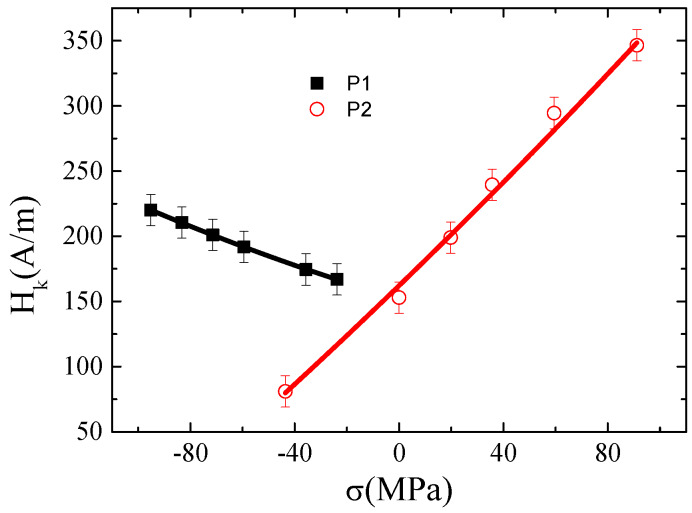
Evolution of anisotropy field, *H_k_*, with applied mechanical stresses, *σ*, for the studied samples. The second-order fitting curve is also shown.

Figure 5 shows the obtained evolution of $H_{K}$ with *σ*. As expected, a polynomial second-order dependence among both parameters is found, $H_{K}=a\sigma^{2}+b\sigma +c$, that is correlated to the magnetostriction constant as follows [26,27]:

(2) $$\lambda_{s}=\lambda_{s0}+k\sigma =\frac{\mu_{0}M_{s}}{3}\;\left(\frac{dH_{K}}{d\sigma }\right)=\frac{\mu_{0}M_{s}}{3}\;\left(2a\sigma +b\right).$$

Values of *λ_s_*_0_ = 1.4 × 10^−7^, *k* = 3.0 × 10^−10^ (MPa^−1^), and *λ_s_*_0_ = −4.6 × 10^−7^, *k* = −2.6 × 10^−10^ (MPa^−1^) for *P_1_* and *P*_2_, respectively, are obtained (where *λ_s_*_0_ is the unstressed valued of the saturation magnetostriction and *k* is the measured slope of the linear dependence of *λ_s_* with stress). Similar results were reported for nearly zero magnetostrictive amorphous alloys (*λ_s_*_0_ ~ 10^−7^ and *k* ~ 10^−10^ (MPa^−1^)) employing the small angle magnetization rotation (SAMR) [27] technique and GSI effect [26].

The described behavior of Z in both samples can be understood in terms of the magnetic anisotropies present in the samples. The amorphous nature of the synthesized ribbons indicates the lack of magnetocrystalline anisotropy. Thus, the presence of a magnetoelastic anisotropy resulting from rapid solidification of the samples (coupling between the quenched-in stresses and *λ_s_*) and the magnetoelastic coupling with the externally applied mechanical stresses determines the magnetic behavior and, consequently, the GMI (or GSI) effect. In the unstressed state, the sample *P_1_* shows an impedance dependence with just one maximum (*SP*), which could be indicative of a not well-defined magnetic anisotropy distribution in the sample, as previously published for other melt-spun ribbons [28]. Furthermore, when axial tensile stresses were applied, the *SP* behavior remains with a noticeable reduction in the impedance values. On the contrary, the application of compressive stresses leads to the induction of a transverse magnetic anisotropy in the sample, clearly visible as the appearance of a double peak (*DP*) in the *Z* curve. Thus, the impedance stress evolution is mainly governed by the positive value of the saturation magnetostriction of the sample. Conversely, the ribbon *P_2_* displays a well-defined transverse magnetoelastic anisotropy for *σ* = 0 MPa, which can only be due to the internal stresses during the cooling down procedure and the coupling with the negative magnetostriction constant (see Figure 4b). This transverse anisotropy is reinforced when tensile stresses were exerted, observing a displacement of *Z* maxima to higher *H* values. The opposite trend is observed for compressive stresses (shift toward lower *H*). Thus, the evolution of the GSI response of the samples (*H* = 0, see Figure 3) is the result of the counterbalance of the internal quenched-in magnetoelastic anisotropy and the magnetoelastic contribution associated with the externally applied stress (tensile or compressive).

It is relevant to mention that, although the magnetostriction constant value of both samples is quite low (nearly zero), the analyzed ribbons can be successfully applied as GSI magnetoelastic sensors, as shown in the next section. Their amorphous structure together with the initial large value of the transverse magnetic permeability, *µ*, favors a large GSI effect that enables the characterization of stresses on the samples through the variations in the ribbon impedance. 

Nevertheless, the previous results permit the election of the optimum ribbon to be employed as the sensor nucleus for the final magnetoelastic devices. On one hand, *P_2_* shows a univocal *Z(σ)* response for −200 MPa < *σ* < 300 MPa at *H* = 0, allowing the determination of both sign and strength (magnitude) of the applied mechanical stress within this interval. However, further application of tensile stresses leads to an indetermination given by the fact that the same *Z* value is observed for different applied tensile stresses on the sample. On the other hand, *P_1_* exhibits a symmetric *Z(σ)* that hinders the determination of the stress sign but favors the univocal characterization when just compressive or tensile stresses are applied.

### 3.2. Magnetoelastic Sensor for Cross-Section Evaluation

**Figure 6:**
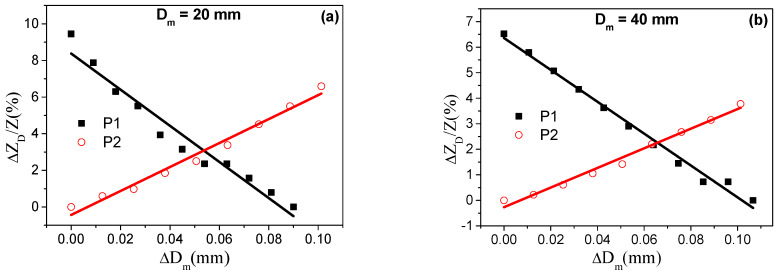
Relative variations of impedance, $\frac{\Delta Z_{D}}{Z}\left(\%\right)$, as a function of the induced variations in probe diameter, Δ*D_m_*, for *D_m_* (**a**) 20 mm and (**b**) 40 mm.

Figure 6 shows the response of both ribbons under the mechanical tests. The main purpose is to analyze their suitability as sensors for the evaluation of the variations in the cross-section dimensions of pieces (see Section 2.2.1). As can be seen, the relative impedance variation ($\frac{\Delta Z_{D}}{Z}\left(\%\right)$) displays, in both analyzed samples, enough sensitivity to measure the micrometric variation of the diameter of the probes (Δ*D_m_*). The effective stress applied to the samples during mechanical tests, *σ*, can be calculated assuming that the strain in the ribbon, *ε*$=\frac{\Delta L_{r}}{L_{r}}$, is the same as the relative changes in the cylinder diameter ($\frac{\Delta D_{m}}{D_{m}}$). Thus, applying Hooke’s law, *σ*, it can be calculated as $\sigma =\varepsilon E_{r}=\frac{\Delta D_{m}}{D_{m}}E_{r}$ [19]. Thus, maximum tensile stresses of 500 and 250 MPa are applied to the samples for *D_m_* = 20 and 40 mm, respectively, assuming *E*_r_ = 100 GPa and Δ*D_m_* = 0.1 mm. Notice that these maximum tensile stresses values are similar to those applied in the initial magnetoelastic characterization (see Figure 3). Thus, according to the Z(σ) curve, the application of tensile stresses, as those applied in this characterization, would give rise to a decrease and an increase in Z for *P_1_* and *P_2_* samples, respectively, as a consequence of the different sign in magnetostriction constant, as stated in Figure 6 (keep in mind that $\frac{\Delta Z_{D}}{Z}\left(\%\right)=\frac{\left|Z\left(D\right)\right|-\left|Z_{MIN}\right|}{\left|Z_{MIN}\right|}\times 100$). 

Moreover, as previously reported [18], the initial bent configuration of the sensing ribbons along the cylindrical probe induces a distribution of tensile and compressive stresses whose strength increases for lower probe diameters. Therefore, this pre-stressed state should display, for *D_m_* = 20 mm, lower impedance variations in comparison with the impedance evolution for *D_m_* = 40 mm. However, as Figure 6 shows, the impedance displays an opposite trend, that is, slightly lower maximum $\frac{\Delta Z_{D}}{Z}\left(\%\right)$ ratios for the larger diameter (*D_m_* = 40 mm). This difference can be explained on the basis of the dependence of the GMI effect on the sample length. In fact, as explained in the experimental section, ribbon lengths (*L_r_* = 5 and 11 cm) were chosen for covering the whole cylinder perimeter ($L_{r}=\pi$*D_m_*). As reported, maximum GMI ratios are found for optimal sample lengths [29]. For the employed samples, this value is around *L_r_* ~ 5 cm. Thus, although, due to initial bending stresses, a lower maximum $\frac{\Delta Z_{D}}{Z}\left(\%\right)$ ratio should be expected for the smallest diameter (*D_m_* = 20 mm), the larger GMI ratio in this shorter ribbon (*L_r_* = 5 cm) dominates the sensor response and provides a higher maximum ratio [19].

Nevertheless, it should be noted that both samples can be employed as sensitive micrometric cross-section sensors, displaying the following sensitivities to the diameter variations: *P_1_* −98 and −62%/mm^−1^; *P_2_* 64 and 38%/mm^−1^ for *D_m_* = 20 and 40 mm, respectively. Notice the highest sensitivity of the *P_1_* sample under both cylinder diameters. Therefore, this larger sensitivity together with the fact that the proposed application is based on the application of just tensile stresses justifies the election of the sample *P_1_* for the design of the proposed magnetoelastic sensors.

**Figure 7:**
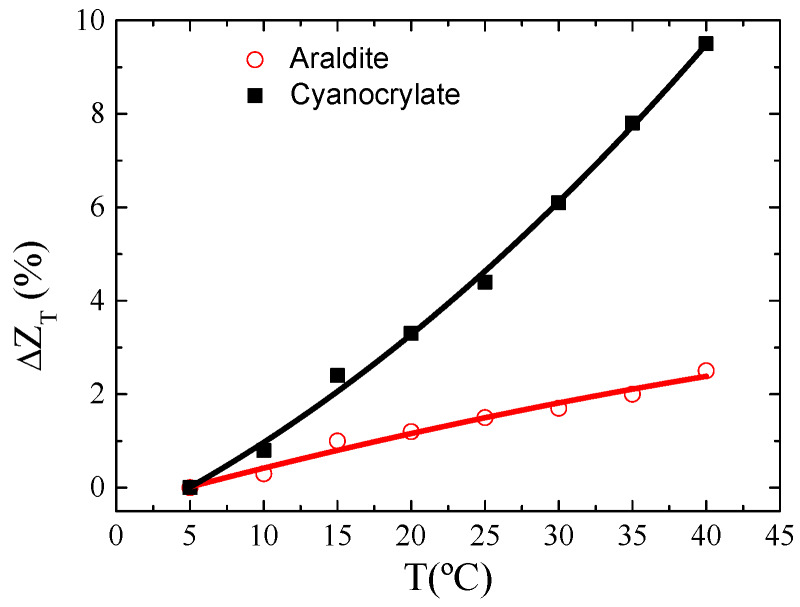
Functional dependence of the relative variation of impedance, $Z_{T}\left(T\right)$, with temperature, *T*.

**Figure 8:**
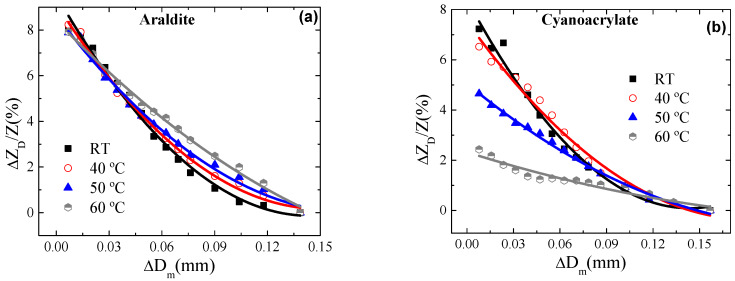
Relative variations of impedance, $\frac{\Delta Z_{D}}{Z}\left(\%\right)$, as a function of the induced variations in probe diameter, Δ*D_m_*, when the sample was fixed with (**a**) Araldite and (**b**) cyanoacrylate. The temperatures were as follows: room temperature, 40 °C, 50 °C, and 60 °C.

Furthermore, to properly characterize the response of the selected *P_1_* ribbon for the proposed magnetoelastic sensor, the mutual effect of temperature and the fixing agent was examined. Its relevance relies on the fact that changes in temperature may alter the properties of the adhesive and, therefore, the transmission of the mechanical stresses together with the impedance itself. At first, the evolution of the relative variation of impedance with temperature was examined fixing the *P_1_* ribbon on a cylindrical methacrylate probe of *D_m_* = 20 mm. This relative variation was normalized employing the expression ΔZT(T)=(Z(T)−Z(T=5℃)Z(T=5℃))×100. As Figure 7 shows, $\Delta Z_{T}\left(T\right)$ is markedly larger when the ribbon *P_1_* was fixed with cyanoacrylate. The best performance employing Araldite was also confirmed during the transmission of mechanical stresses. Figure 8 shows $\frac{\Delta Z_{D}}{Z}\left(\%\right)$ for the mechanical tests performed at different temperatures. As Figure 8 shows, no relevant changes in $\frac{\Delta Z_{D}}{Z}\left(\%\right)$ are observed when the mechanical tests were performed at different temperatures with Araldite. Maximum differences in $\frac{\Delta Z_{D}}{Z}\left(\%\right)$ around 1.5% are obtained when measuring Δ*D_m_* at different temperatures (i.e Δ*D_m_* = 0.1 mm). However, the cyanoacrylate adhesive strongly affects the behavior of the impedance, showing significant changes in the maximum diminution of $\frac{\Delta Z_{D}}{Z}\left(\%\right)$ with Δ*D_m_* (from 7.2% to 2.4% at room temperature and 60 °C, respectively), invalidating its use in these magnetoelastic sensors. These results are in good concordance with Figure 7. This effect could be related to the higher coefficient of thermal expansion of the cyanoacrylate. Thus, when temperature changes, the larger variations of dimensions displayed by cyanoacrylate may exert more stresses on the ribbon, leading to changes in Z even under zero applied external stresses.

As the main conclusion, it can be pointed out that the proposed magnetoelastic sensor based in soft magnetic (nearly zero magnetostrictive) amorphous ribbons demonstrated its capability to characterize micrometric variations of the cross-section dimensions. This sensor prototype exhibits several advantages such as high Young modulus and tensile strength, parameters that favor an accurate stress characterization. Furthermore, these amorphous alloys can be easily adapted to the whole perimeter of the pieces to characterize them even if they offer an irregular geometry and/ or small size. In this sense, although the employed probes in this analysis displayed a smooth and regular surface, the proposed magnetoelastic sensor was successfully employed in systems with irregular and rough surfaces [30]. Precisely, this adaptability permits integrating the stresses around the whole perimeter, enabling us to obtain a mean variation of the cross-section (even for irregular shapes) where all points around the piece perimeter can contribute to the general stress characterization. In contrast, piezoresistive strain gauges (see Figure 1a) can only compute the stresses in a local point, hindering the mean estimation along the whole perimeter and favoring the loss of information especially in irregularly shaped pieces.

### 3.3. Development of A Flow Meter

The magnetoelastic ribbon was located perpendicularly to the water flow inside the pipe (see Section 2.2.2) which exerted variable bending (tensile) stresses on the ribbon. The principle of operation of the proposed flow meter relies on the changes of impedance experimented by the *P_1_* ribbon when the water flow changes. To obtain significant variations of *Z*, the sample required a certain pre-tightened state; otherwise, no variations of impedance were observed. 

**Figure 9:**
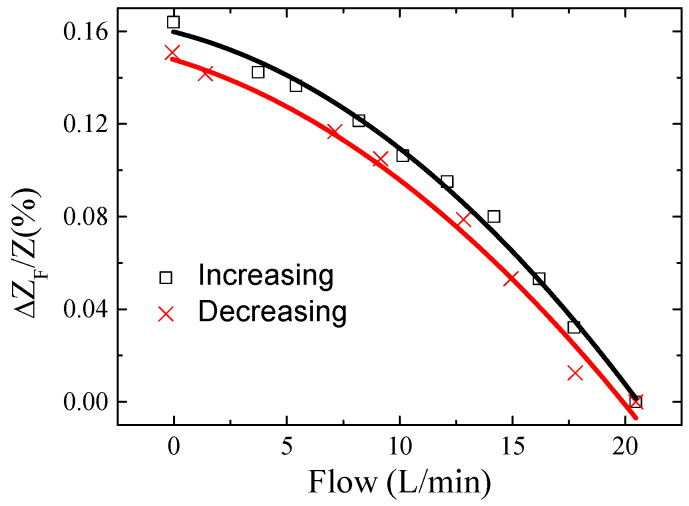
Relative variations of impedance, $\frac{\Delta Z_{F}}{Z}\left(\%\right)$, as a function of the water flow.

The relative variations of impedance, $\frac{\Delta Z_{F}}{Z}\left(\%\right)$, were studied as a function of the water flow in the pipe. In order to study reproducibility and hysteretic behavior of the sensor response, the water flow was gradually increased and subsequently decreased, repeating this process in different attempts. The impedance changes were registered simultaneously with the controlled variation of the water flux, measured by the commercial dispositive. As an example, Figure 9 shows $\frac{\Delta Z_{F}}{Z}\left(\%\right)$ versus water flow for a scan of measurements. It can be seen that, despite the low impedance variations, the changes in the water flow can be properly determined. Since no linear response was obtained, a standard sensitivity cannot be calculated. In consequence, the sensor response was fitted to a polynomial second-order curve ($\frac{\Delta Z_{F}}{Z}\left(\%\right)=a+bF+cF^{2}$, where *F* is the water flow) for the set of performed measurements, both increasing and decreasing *F*.

Table 1 shows the mean values of the estimated polynomial second-order parameters, together with the standard error of the mean. It should be pointed out that there is a slightly higher uncertainty for the decreasing flux measurements. Concerning the sensor hysteresis, only small differences can be observed in impedance evolution under increased and decreased water flow. To quantify this effect, a “hysteretic coefficient (*HC*)” can be calculated. This parameter can be defined as the relative maximum variation of the magnitude output (in this case, $\frac{\Delta Z_{F}}{Z}\left(F\right)$) between increasing and decreasing water flow changes. In this case, as Figure 9 shows, the relative variation of the *a* parameter (*F* = 0) can be employed in the hysteresis estimation. Thus, a value of $HC=\left(\frac{a_{Inc}-a_{Dec}}{a_{Dec}}\right)\times 100\approx 9\%$ is obtained.

Eventually, it is also relevant to note that the suggested sensor can be employed not only for water flow measurements, permitting the generalization of its employment for other liquids. Finally, in comparison with other flow meter devices, the main advantages of the proposed sensor rely on its low cost and its capacity of being scalable to different pipe diameters and sizes, thereby broadening its applicability. However, some future work should be addressed, such as the design of a holder for fixing the sensor firmly inside the pipe, an analysis of the lifetime of the sensor based on the number of cycles with a linear response, and the effect of aging on its response.

## 4. Conclusions

The GSI response of two nearly zero magnetostrictive ribbons with positive (*P_1_*: (Co_0.93_ Fe_0.07_)_75_ Si_12.5_ B_12.5_) and negative (*P_2_*: ((Co_0.95_ Fe_0.05_)_75_ Si_12.5_ B_12.5_) magnetostriction constant was analyzed for their potential application as a sensor nucleus in magnetoelastic sensors. 

Due to the absence of any other magnetic anisotropy, the magnetoelastic anisotropy determines the magnetoelastic response of the samples and particularly the GSI effect. Under zero applied magnetic field, the *P_1_* sample with positive magnetostriction showed a maximum of Z(σ) in the unstressed state. On the contrary, the presence of a transverse magnetoelastic anisotropy, as a consequence of the negative magnetostriction of P_2_, leads to the appearance of a maximum in Z(σ) after the application of stresses.

From the applied point of view, despite the low magnetostriction constant (~$10^{-7}$), both samples are capable of characterizing the applied stresses. However, different behaviors at null magnetic field were observed. The sample *P_2_* permits the determination of both the sign and the intensity of the applied mechanical stresses within a certain interval. Nevertheless, further application of tensile stresses may lead to an indetermination; the same value of impedance can be obtained at different tensile stress intensities. On the contrary, the larger sensitivity and symmetric Z(σ) behavior of *P*_1_ allows univocal determination when only compressive or tensile stresses are involved. Nevertheless, the sign of the stresses cannot be determined.

Based on its properties, two different magnetoelastic (cheap and adaptable) sensors were designed employing the P*_1_* ribbon as a sensor nucleus. One was for the characterization of the variation in the cross-section of pieces that is adaptable to irregular and/or rough surfaces and allows the estimation of the mean stresses along the whole perimeter of the section, and the other was for the measurement of the liquid flow inside a pipe (flow meter).

## Figures and Tables

**Table 1:** Polynomial second-order fitting parameters of the sensor response (mean values and standard error).

Mean Fitting Parameters	*a* (%)	*b* (%min/L)	*c* (%min^2^/L^2^)
Increasing	0.145 ± 0.009	−0.0025 ± 0.0004	−(2.4 ± 0.2) × 10^−4^
Decreasing	0.133 ± 0.009	−0.0027 ± 0.0009	−(2.1 ± 0.5) × 10^−4^
